# A one health analysis of the reemergence of dog-mediated human rabies in Peru

**DOI:** 10.1038/s41598-026-65032-3

**Published:** 2026-07-31

**Authors:** Ricardo Castillo-Neyra, Lizzie Ortiz-Cam, Elvis W. Díaz, Jorge L. Cañari-Casaño, Sergio E. Recuenco, Valerie A. Paz-Soldán

**Affiliations:** 1 Department of Biostatistics, Epidemiology and Informatics, Perelman School of Medicine, University of Pennsylvania, Philadelphia, 423 Guardian Drive, Room 715, Blockley Hall, Philadelphia, PA 19104-6021 USA; 2 Department of Pathobiology, School of Veterinary Medicine, University of Pennsylvania, Philadelphia, PA USA; 3 One Health Unit, School of Public Health and Administration, Universidad Peruana Cayetano Heredia, Lima, Peru; 4 Department of Tropical Medicine and Infectious Disease, Celia Scott Weatherhead School of Public Health and Tropical Medicine, Tulane University, New Orleans, LA USA; 5 Department of Preventive Medicine and Public Health, Facultad de Medicina San Fernando, Universidad Nacional Mayor de San Marcos, Lima, Peru

**Keywords:** Neglected tropical disease, One health, Outbreak, Rabies, Swiss cheese model, Zoonoses, Diseases, Ecology, Ecology

## Abstract

The 2023 reemergence of dog-mediated human rabies in Arequipa, Peru, after nearly a decade of absence, represents a critical failure in public health systems. Despite regional progress toward the “Zero by 30” strategy, this human rabies case highlights persistent systemic vulnerabilities. Here we investigate how this preventable death emerged, by integrating epidemiologic, socioecological, and policy data through a One Health lens. Using the Swiss cheese model, we identified multiple defensive layers—policies, outbreak response, system capacity building, and socio-geographical features—that aligned to permit transmission. We identified critical breaches, such as an 80% decrease in surveillance sampling, insufficient management of feral dog populations in urban corridors, and clinical misdiagnosis of the victim. These findings suggest that rabies persists not for lack of tools, but due to fragmented application and institutional amnesia. We propose a redesigned multisectoral framework to transform human rabies from a neglected tragedy into a true “never event”.

**Supplementary Information:**

The online version contains supplementary material available at https://doi.org/10.1038/s41598-026-65032-3.

## Introduction

Dog-mediated human rabies remains a major public health threat, causing tens of thousands of deaths annually around the world despite being entirely preventable^1^. While Latin America has reduced incidence under the "Zero by 30" strategy^2^, persistent enzootic foci in countries like Peru complicate elimination^3,4^. By 2006, 88% of Peru was declared rabies-free; however, between 2009 and 2015, dog rabies reemerged in several regions, including the department of Arequipa^3,4^, highlighting the sustained circulation of the virus despite ongoing vaccination campaigns. While human rabies cases have become rare in the country, 395 dog rabies cases have been detected in Arequipa city since 2015^5^, and the system failed to prevent the first human death in 2023.

This 2023 death was not a random occurrence, which results from the gradual erosion of systemic defenses. Traditional public health models often view such tragedies through a narrow clinical or veterinary lens, focusing on individual vaccine failures or bite incidents^6–10^. However, the reemergence of human rabies suggests a deeper breakdown in One Health governance^11^. This breakdown is characterized by a decoupling of policy from local socio-ecology^12–16^, where institutional efforts fail to account for the complex interactions between human behavior, dog ecology, and a fragmented health care infrastructure.

To understand the mechanics of this system failure, we adopt a One Health framework^17^, which allows for a comprehensive mapping of critical interconnections between human health, animal reservoir dynamics, and environmental factors. In the context of rabies, the One Health approach is essential to visualize how institutional fragmentation and the disarticulation of multisectoral efforts create systemic vulnerabilities^11,17^. By integrating eight years of longitudinal data (2015–2023), including canine surveillance records, mass vaccination coverage, dog ecology tracking, and qualitative stakeholder mapping, we reconstruct the “holes” in Arequipa’s public health system.

Our study moves beyond the “what” of the 2023 incident to investigate the “why”. We analyze socio-ecological elements, such as the dry water channels that facilitate dog movement, to multifaceted barriers to dog vaccination, to what we interpret as a pattern consistent with “institutional amnesia”—a documented decline in surveillance intensity during periods of low human incidence. By examining this case through a One Health lens, we provide a blueprint for identifying and plugging systemic vulnerabilities in rabies elimination programs, offering critical lessons for the global effort to reach zero deaths by 2030.

## Methods summary

Our mixed-methods retrospective case study integrates epidemiological surveillance data (2015–2023), repeated door-to-door household surveys (up to 8000 households annually), GPS-based dog ecology data, seven focus group discussions (n = 56), and a Group Model Building workshop with 69 stakeholders. We applied the Swiss cheese model as an analytical framework to identify structural vulnerabilities across four defensive layers: policies, outbreak response activities, system capacity building, and socio-geographical features. Stakeholder mapping was used to characterize governance fragmentation. Full methodological details are provided in the Methods section.

## Results

### The incident: a chronology of system failure

**Fig. 1:**
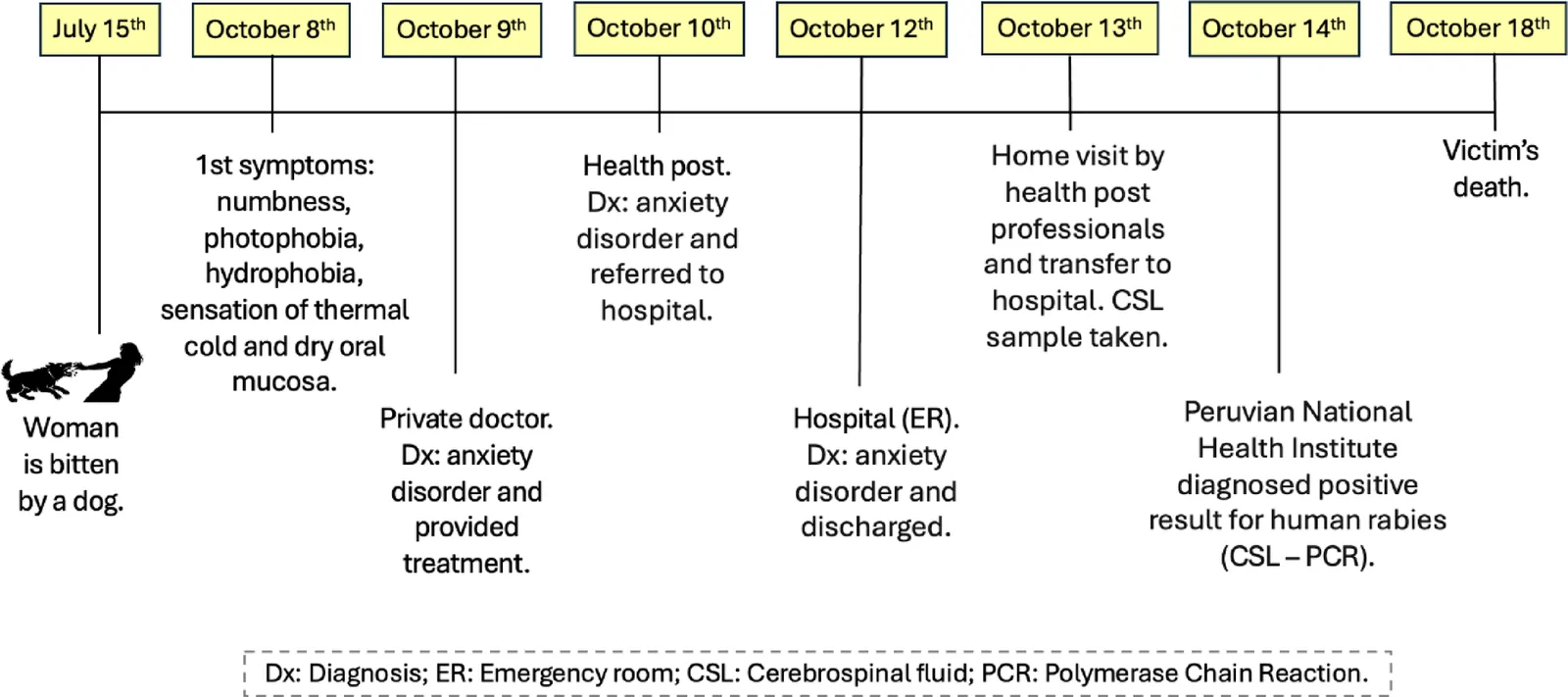
Human rabies case timeline, Arequipa 2023^20–22^.

The 2023 human rabies case occurred in a context of documented systemic vulnerabilities, which in retrospect we interpret as consistent with a predictable outcome rather than a chance event. The victim resided in Chiguata, a peri-urban area characterized by rapid growth and active canine rabies transmission. On the night of July 15, 2023, a female resident of the peri-urban district of Chiguata was bitten on the upper extremity by a free-roaming dog (Fig. 1). The incident occurred in the victim’s backyard when the dog breached the perimeter fence to prey upon the victim’s poultry; the victim was bitten while trying to repel the dog with her bare hands^18^. The animal left the premises immediately after the incident and could not be quarantined or tested for rabies. The victim neither reported the bite nor sought post-exposure prophylaxis (PEP), consistent with previously documented patterns of low rabies risk perception within the community^19^. Approximately 85 days later, on October 8th, the victim developed prodromal symptoms including localized numbness, photophobia, hydrophobia, and altered thermal sensation, suggesting a prolonged incubation period during which no preventive intervention occurred (Fig. 1). As neurological symptoms progressed and medical care was sought, the initial clinical presentation was misattributed to generalized anxiety and psychiatric disorders rather than rabies^20–22^: the victim never reported the dog bite, but she was also not asked.

By the time rabies was considered and laboratory confirmation obtained, the disease had progressed irreversibly, resulting in the victim’s death^20–22^. Figure 1 illustrates that the victim had multiple subsequent encounters with separate healthcare facilities between October 10th and 12th. During these visits, persistent warning signs were repeatedly attributed to anxiety rather than considered in the differential diagnosis of rabies, delaying definitive PCR testing until the patient was critically ill. It is important to note that Health post professionals do not routinely conduct home visits unless a dog bite has been formally reported, which in this case, it had not. The specific trigger that prompted the eventual home visit and raised clinical suspicion for rabies remains undocumented in the available records.

**Fig. 2:**
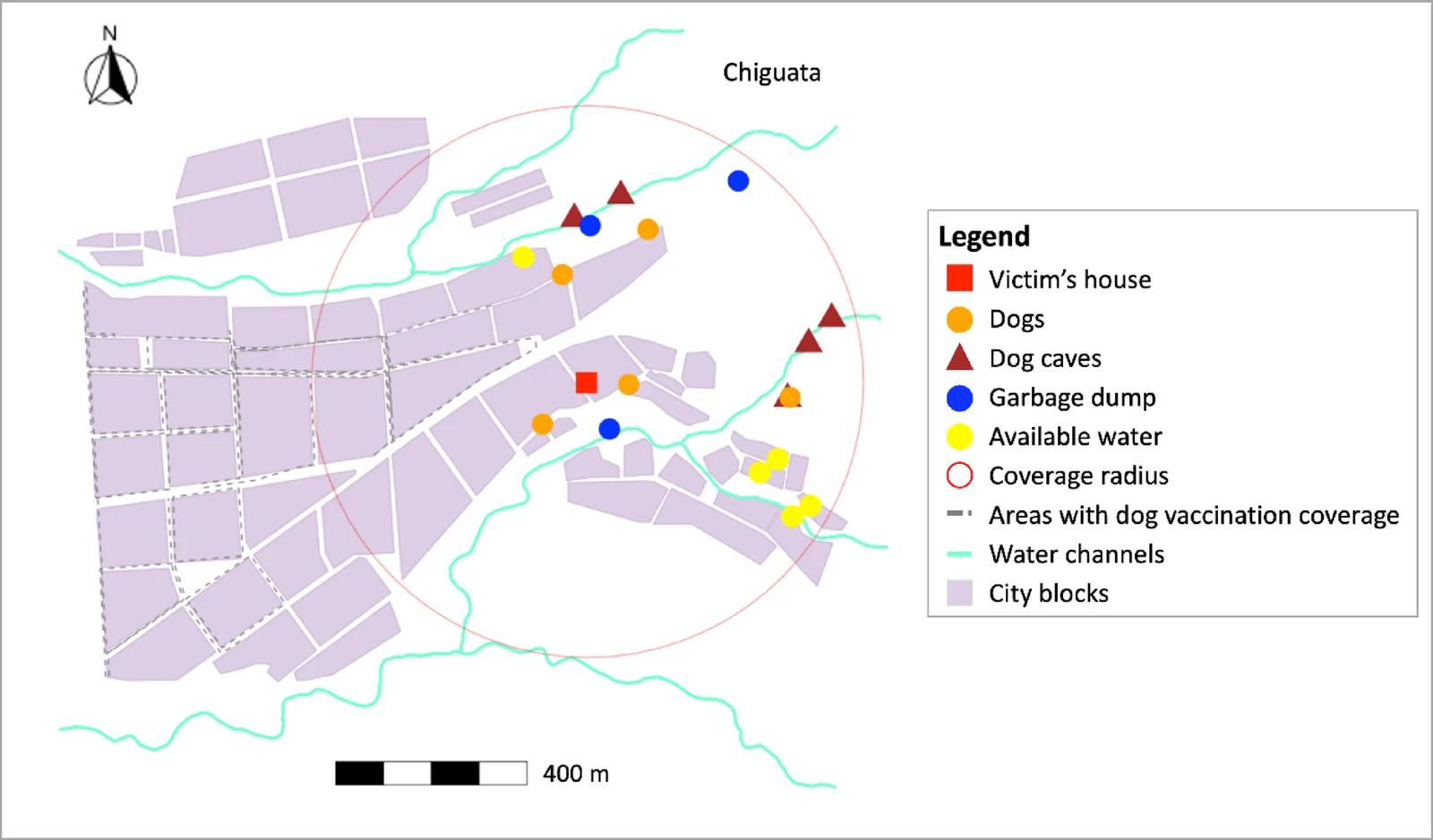
Study population characteristics.

The spatial overlay presented in Fig. 2 demonstrates that the victim’s household was situated directly at the intersection of unmanaged water channels, active garbage dumps, and documented feral dog caves. Critically, Fig. 2 highlights that although a subset of municipal blocks fell within the formal 400-m coverage radius of scheduled vaccination campaigns, the complex physical topography and nearby open garbage dumps directly incentivized the clustering of free-roaming dogs reservoirs, thereby overriding the protective effects of localized block-level domestic vaccination.

### Systemic breaches: the Swiss cheese model analysis

**Fig. 3:**
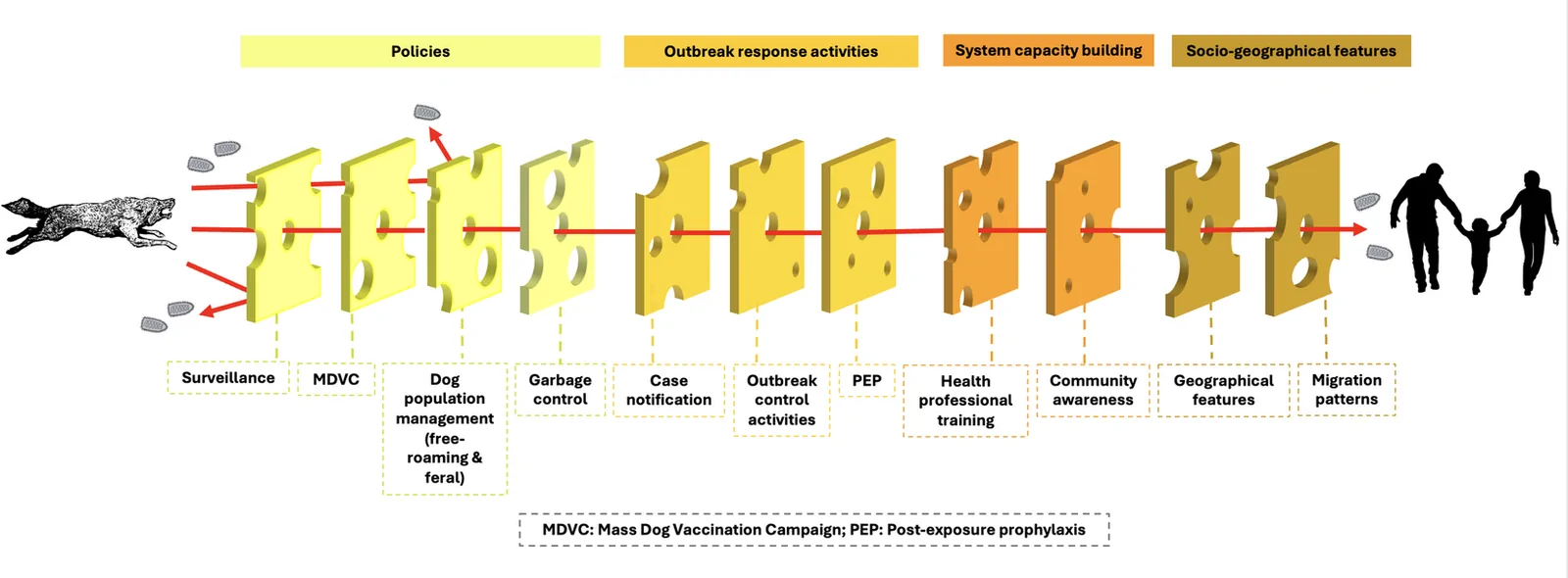
Swiss cheese model analysis for dog-mediated human rabies.

To understand how multiple breaches in the system aligned to cause a catastrophic event, we applied the Swiss cheese model (Fig. 3) for a multi-layers analysis of the situation. Each layer in our model represented a component of the rabies prevention system, including dog vaccination programs, epidemiological surveillance, access to post-exposure prophylaxis (PEP), professional training, and community awareness. We identified the specific “holes” within each layer, which represent structural or operational weaknesses. The alignment of these vulnerabilities created a pathway through which the virus was able to reach a human host.

#### Policy gaps

**Fig. 4:**
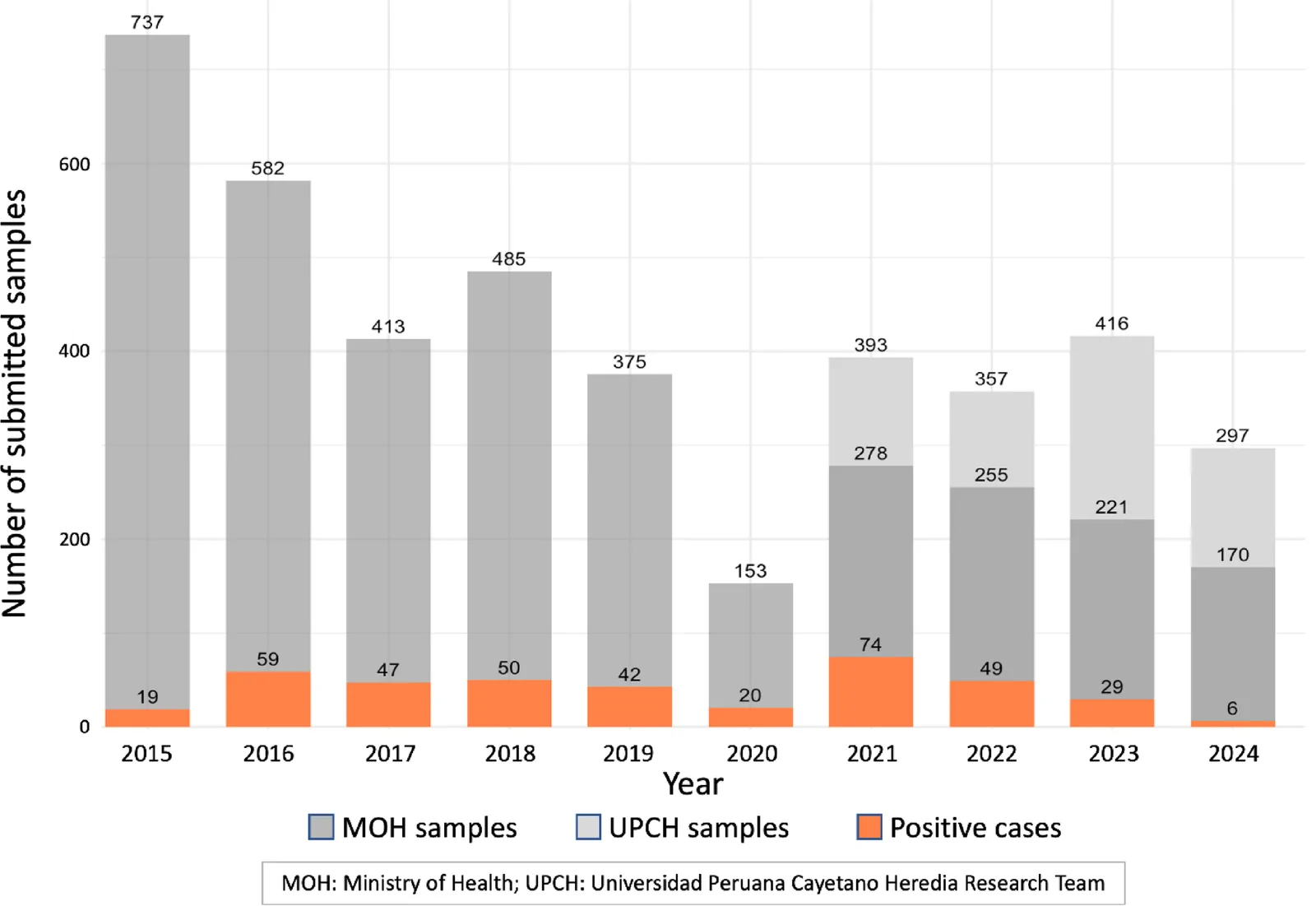
Dog rabies cases identified and submitted samples in Arequipa City, 2015 to August 2024.

The sharp divergence between the high number of confirmed canine cases and the precipitous 80% drop in submitted diagnostic samples between 2015 and 2020 suggests that underdetection was driven by an active reduction in collection efforts rather than a natural decline in viral circulation (Fig. 4). underdetection of virus. This interpretation is directly supported by the subsequent case spike observed when sampling intensities were partially restored (Fig. 4), confirming that trends from inconsistent surveillance misrepresent true field epidemiological dynamics. Concurrently, vaccination planning for the entire city relied on an outdated Human-to-Dog (H:D) ratio of 10:1, whereas our door-to-door surveys established a more accurate ratio of 6:1^23^, indicating a systemic underestimation of the dog population targeted for vaccination.

More specifically, the H:D ratio used by the Chiguata heath network showed high inter-annual variability, ranging from 2.5 to 3.4 across consecutive years (Table 1), suggesting that denominator estimation was methodologically unstable and therefore an unreliable basis for longitudinal coverage tracking.This instability is reflected in reported MDVC coverage exceeding 100% in Chiguata (113.2% in 2022, Table 1), a finding consistent with at least three non-mutually exclusive explanations: (1) systematic underestimation of the dog population denominator, as supported by our survey data^23^S; (2) double-counting of vaccinated animals across campaign days or geographic units, a known operational challenge in mass vaccination campaigns; and (3) vaccination of dogs from neighboring districts during campaign events, inflating the numerator beyond the local target population.

**Table 1:** Estimates used by Chiguata health network for local public health activities (2021–2023).

Year	Human population	Ratio H:D	Dog population	Vaccinated dogs	Mass dog vaccination campaigns (MDVC) coverage
2021	6208	3.4	1800	1500	83.3%
2022	3266	2.5	1306	1478	113.2%*
2023	4629	2.5	1852	1269	68.5%

Source: Arequipa—Caylloma Health *Red* (Network). September 2023.

*More than 100% coverage was reported, which indicates an underestimation of the canine population.

#### Outbreak response

Timeliness of outbreak detection represented an additional vulnerability within the rabies prevention system. Previous analyses indicate that approximately 62% of rabies cases in the region suffered from late reports^24^, delaying the effectiveness of reactive control measures such as ring vaccination, enhanced surveillance and post-exposure prophylaxis (PEP) administration. Community members sometimes clandestinely buried rabid dogs to avoid social stigma^25^, further masking the outbreak’s extent, a pattern documented consistently across our community focus groups, in which participants described fear of community judgment and distrust of official responses as primary reasons for concealing animal deaths.

#### System capacity building

**Fig. 5:**
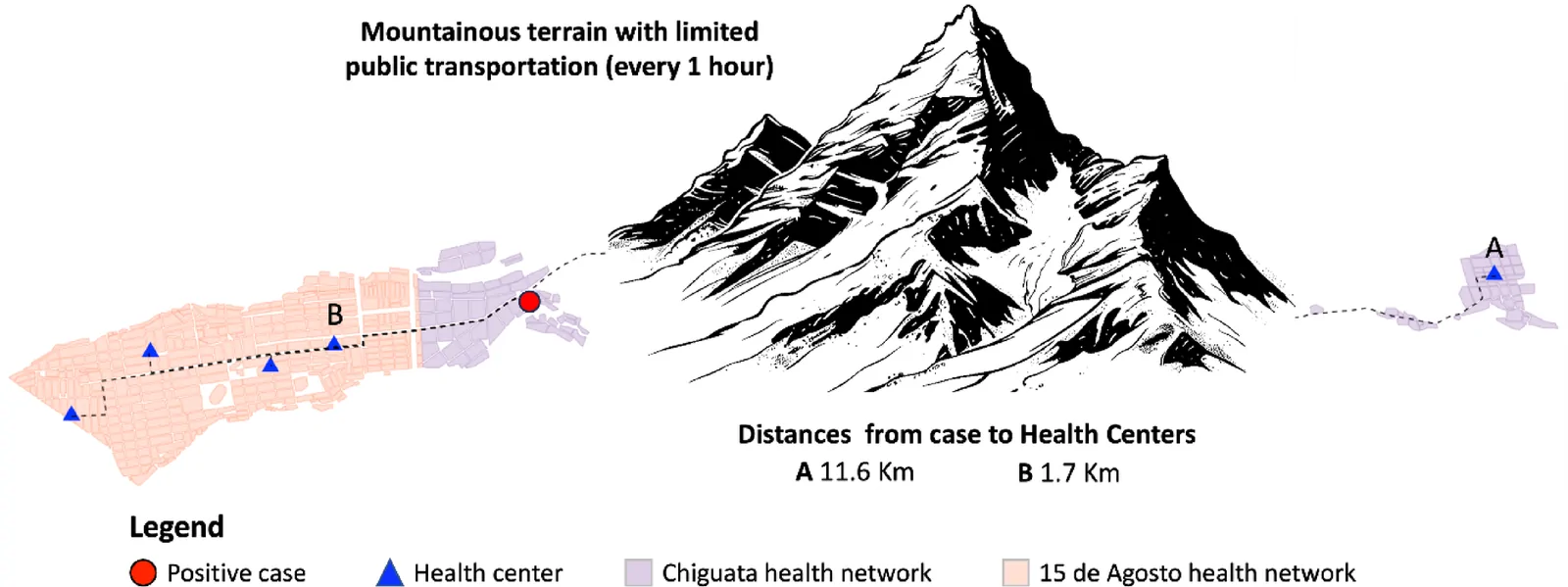
Distribution of health centers in health networks, and distance from the case residence to those centers.

Limited training in rabies recognition is consistent with animal exposure not being routinely investigated. Simultaneously, the absence of a structured community awareness plan in Chiguata likely contributed to residents underestimating risk^3,25^. This is compounded by the geographic “distance to care”: the Chiguata health post had no rabies vaccine at the time of the incident; the victim was referred to a different health facility due to jurisdictional divisions, 11 km away, creating delays and confusion. Critically, this distance was not traversed on flat urban roads: Chiguata’s peri-urban geography means that reaching the nearest facility with PEP required crossing mountainous terrain, substantially increasing travel time and physical hardship. Figure 5 illustrates the distribution of health centers and the significant travel distances from the victim’s residence to specialized care, highlighting both the geographic remoteness and the topographic barriers that may have delayed timely access to PEP. Additionally, distrust of official vaccination teams, rooted in prior experiences of abrupt campaign cancellations and inconsistent follow-up, contributed to sub-optimal MDVC participation and delayed care-seeking following exposure^19^.

#### Socio-geographical features

**Fig. 6:**
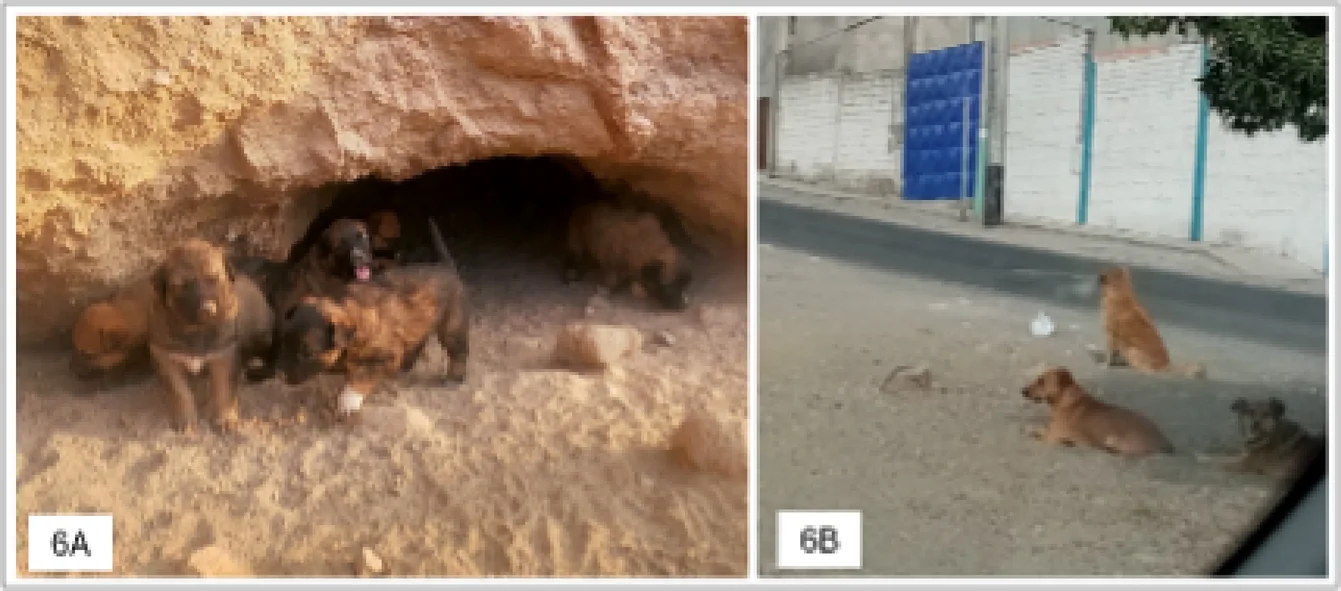
Dog population in Arequipa, Peru. (**A**) Caves used by feral dogs. (**B**) Presence of free-roaming dogs in the city. Photographs by Universidad Peruana Cayetano Heredia (UPCH) research team, 2023.

The dry water channels in the city serve as efficient corridors for feral and free-roaming dogs to travel between urban and peri-urban zones^15^, providing them with a food source and refuge (Fig. 1S). Our field observations (Fig. 6) document the active presence of feral dog populations inhabiting caves (6A) and free-roaming dogs in the area (6B). The water channels are therefore relevant not as a direct causal pathway in this specific case (direct contact between the confirmed rabid biting dog and the feral populations inhabiting these corridors cannot be established from available records), but as a structural feature of the peri-urban landscape that sustains an unvaccinated dog reservoir^26^. Moreover, previous studies reported seasonal human and animal migration between Arequipa and Puno, which likely contributed to the reintroduction of the virus prior to its eventual detection in 2015^27^. In Chiguata, field observations indicated high household mobility and frequent recent immigration, patterns previously associated with differences in dog ownership, vaccination practices, and health-seeking behaviours compared to long-term residents^13,25^. These dynamics may create pockets of lower vaccination coverage and delayed care-seeking, increasing vulnerability to rabies reemergence.

### Stakeholder fragmentation and intersectoral conflicts

**Fig. 7:**
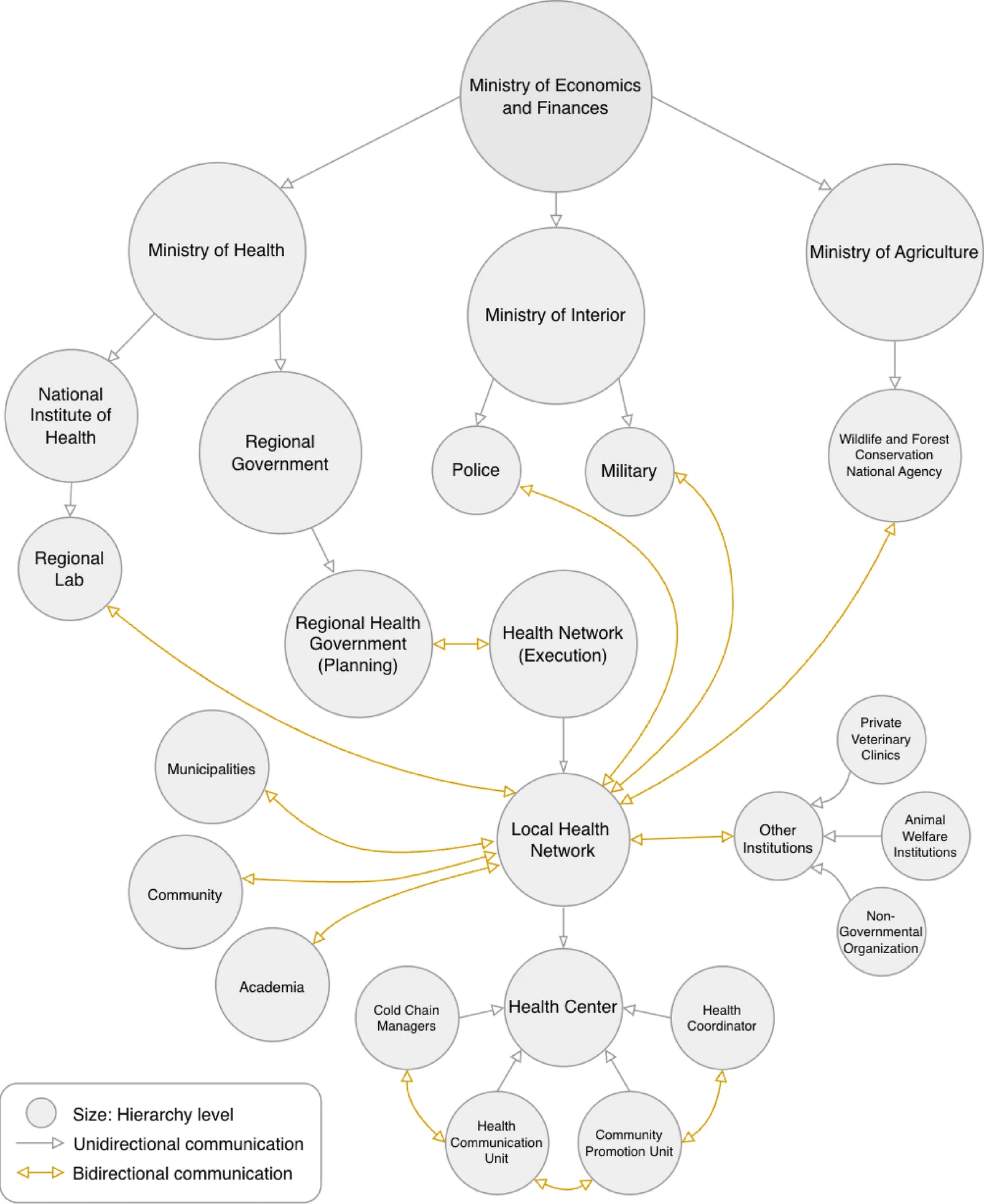
Stakeholder map for dog rabies elimination in Peru.

The stakeholder mapping (Fig. 7) is consistent with a governance structure in which fragmentation contributes to the conditions enabling the case. Rabies control activities in Peru involve multiple actors operating at different levels, including national ministries, regional health authorities, municipal governments, academic institutions, and community organizations. However, relationships between these actors are characterized by rigid sectoral silos and a distinct lack of horizontal coordination, creating “missing links” between critical sectors.

National-level institutions, for instance the Ministry of Economics and Finances emerges as a highly influential but operationally distant stakeholder; while it does not participate directly in rabies control activities, it exerts substantial influence through budget allocation and financial authorization, shaping the scale and continuity of interventions. The Ministry of Health (MOH) and the Regional Health Government serve as the primary decision-makers, controlling vaccine procurement and clinical guidelines^28,29^. However, our stakeholder mapping shows that MOH is highly vertical. Information flows upward to the national level, but there is a significant “bottleneck” in horizontal data sharing with municipal authorities who manage the local environment.

While the MOH dictates rabies policy, the actual management of “upstream” risk factors, such as solid waste management, control of dog populations, and regulation of spaces like the water channels*,* falls to local municipalities and district governments. Our analysis identified a critical lack of formal coordination between health sectors and these municipal actors^19^. This disconnection creates a scenario where dogs may be vaccinated in one sector while the environmental conditions that allow feral dogs to thrive in the water channels remain undressed, undermining the overall effectiveness of the elimination strategy. We also identified key stakeholders with high potential influence but low current involvement, specifically the National Forestry and Wildlife service^30^ and local Police/security forces^31^. Despite their role in animal welfare and public safety, they remain on the periphery of the rabies elimination program. Their exclusion from active surveillance represents a missed opportunity for a cohesive One Health response that includes wildlife-urban/peri-urban interfaces and enforcement of dog-leash laws.

Academia provides high-interest support through research and surveillance assistance. However, their role is often limited to technical support rather than policy-making. We also identified that local non governmental organizations (NGOs) provide support through promotion and activities that generate trust within the community. On the other hand, the mapping further highlights a distrust gap between community members and official health promotion teams, which directly contributed to the clandestine burial of rabid dogs, bite victims’ delay in seeking care, and dog owners’ distrust of the MOH vaccine. We categorized stakeholders based on their influence, hierarchy, and type of communication (unidirectional or bidirectional) and presented in Fig. 7.

## Discussion

Our study is consistent with the interpretation that the 2023 human case was facilitated by a One Health governance gap; and it serves as a stark reminder that regional progress toward the “Zero by 30”^2^ strategy is fragile and easily reversed. The stakeholder map (Fig. 7) highlights that rabies persists not for a lack of effective technical tools, but due to an institutional misalignment where hierarchy (MOH/Ministry of Economics and Finances) is decoupled from local jurisdiction (Local Health network, Health networks, and Municipalities). To transition from a reactive posture to successful elimination, Peru must move beyond vertical, health-centric interventions and establish a horizontal governance framework that integrates apparently peripheral allies into the center of the response. Such multisectoral coordination is essential for managing the free-roaming and feral dog reservoirs that currently bypass traditional vaccination boundaries.

The structural fragmentation documented in the stakeholder map is compounded by a temporal decay in surveillance. Between 2015 and 2020, Arequipa’s official surveillance sampling decreased by nearly 80% (Fig. 4), a trend consistent with canine rabies circulating with reduced detection capacity during this period. This decline—occurring in the absence of human cases and coinciding with competing health priorities—is consistent with what the literature describes as institutional amnesia^32,33^: the gradual erosion of political and financial commitment to disease control during inter-outbreak periods^5,34^, regardless of ongoing transmission risks in the animal reservoir^4,35,36^. We present this not as an empirically demonstrated mechanism, but as a plausible interpretive framework that our surveillance trend data support but cannot confirm, and that warrants prospective investigation. The methodological instability in dog population denominators further undermined the reliability of coverage metrics as indicators of true population immunity, compounding the detection gap created by the surveillance decline.

The clinical progression of the 2023 case underscores a critical breach in the System Capacity Building layer of our Swiss cheese model. During the victim’s multiple health center visits, the failure to include rabies in the differential diagnosis resulted in symptoms being misattributed to psychiatric disorders^20–22^. This diagnosis gap is consistent with reduced clinical suspicion among providers and a systemic failure to routinely inquire about animal exposure in peri-urban settings where dog bites may be underreported. Without continuous training that survives the inter-outbreak periods, the health system risks remaining reactive rather than preventive, transforming a treatable exposure into an irreversible tragedy.

Our findings challenge the assumption of Peru’s centralized rabies control program, which largely focuses on owned dogs^26^ and assumes unowned populations are negligible. The water channels of Chiguata act as unmonitored ecological corridors that link feral dogs to urban households, facilitating undetected virus movement^15^. Current policies fail to address these One Health bridges because responsibility is fragmented: the MOH manages vaccines, while local municipalities are responsible for the environmental factors and dog population management^37^. In addition, no sector is clearly responsible for feral dog control. This fragmentation prevents a cohesive response to the complex socio-ecological reality of rabies, especially in peri-urban areas^12–16^.

To achieve “Zero by 30”, surveillance must transition from a retrospective reporting tool to a proactive early warning indicator. Strengthening bite registries and integrating primary-care data are the simplest and most informative ways to guide efficient PEP allocation. This integration is best operationalized through Integrated Bite Case Management (IBCM)^17^, a One Health framework that facilitates real-time communication between clinical providers and veterinary services. By investigating the health status of the biting animal, IBCM significantly increases the probability of identifying rabid animals while reducing the unnecessary administration of PEP in low-risk exposures. In Arequipa, the adoption of such a framework would bridge the current implementation gap, transforming passive bite reporting into an active early-warning system capable of interrupting transmission chains before human fatalities occur. Geographic access to care represents an additional barrier that IBCM alone cannot resolve: the mountainous topography between Chiguata and the nearest PEP-equipped facility means that the effective travel burden—in time, cost, and physical effort—is considerably greater than the linear distance implies, a dimension of geographic inequity that standard health facility mapping tools typically fail to capture. However, the success of these technical systems depends on community risk perception, which is often overlooked. The normalization of dog bites and the practice of clandestinely burying rabid animals to avoid stigma, documented in our focus groups, underscore the importance of sustained, community-embedded awareness strategies, as opposed to campaign-only communication efforts that lose reach during inter-outbreak periods.

Our analysis had some limitations. Surveillance data may underestimate the true burden of canine rabies due to underreporting or variations in diagnostic capacity over time. Estimates of dog population size and vaccination coverage are subject to uncertainty, particularly in rapidly expanding peri-urban areas where dog populations may change quickly. Methodologically, the Swiss cheese model provides a conceptual representation of systemic failure but does not quantify the relative contribution of each layer of vulnerability.

A human death from dog-mediated rabies in a setting with over a decade of active control infrastructure constitutes what patient safety science terms a ‘never event’^38^—an occurrence so serious and so preventable that a single case demands rigorous system analysis, not as an anomaly to be explained away, such an event serves as clear evidence of systemic failure requiring immediate correction action. We therefore offer our findings as the identification of systemic vulnerabilities revealed through our One Health analysis. We present the mixed methods, tools, and frameworks used by our team, including stakeholder mapping and the Swiss cheese model, to potentially guide other researchers and program managers as they analyze similar events within their own institutional, ecological, and social context. While some conclusions are specific to Arequipa, the full impact of local factors (e.g. governance fragmentation, community risk perception, and the ecological role of water channels) will only become clear as additional cases emerge.

Ultimately, the 2023 case is consistent with the interpretation that progress in isolated components, such as laboratory capacity or vaccine procurement, cannot guarantee protection if the system is not aligned across sectors and time. Eliminating human rabies deaths requires treating the disease explicitly as a One Health governance challenge^11,17^. Sustained interruption of transmission depends on stable political commitment that persists in the absence of human cases^39–41^, ensuring that surveillance, animal ecology, and community engagement are jointly managed to make rabies a true “never event”.

## Methods

### Study design and analytical framework

We conducted a mixed-methods retrospective case study integrating epidemiological, ecological, and qualitative data to investigate the systemic factors that enabled the occurrence of a human rabies case in Arequipa, Peru in 2023. The analysis was guided by a One Health framework, which conceptualizes rabies transmission as the product of interactions among human, animal and environmental systems. To operationalize this perspective, we applied the Swiss cheese model^42^, a framework traditionally used in high-risk industries^43^ to examine how multiple layers of defense can be breached when structural weaknesses align.

Faced with the challenge of organizing multilevel findings into a coherent analytical structure, our research team evaluated several frameworks and determined that the Swiss cheese model best captured the layered, interdependent nature of the system vulnerabilities identified in our data. The four defensive layers used in the analysis (policies, outbreak response, system capacity building, and socio-geographical features) were not imposed a priori from the literature, but emerged inductively through an iterative team process: researchers from epidemiology, veterinary medicine, social science, and public health engaged in repeated structured discussions to identify the main thematic groupings that best represented the data, subsequently mapping these onto the Swiss cheese model architecture. Once the layers were defined, each was operationalized using pre-specified indicators derived from the literature and from Peru’s national rabies control guidelines^26^. A ‘hole’ was defined as a documented, measurable deviation from the standard of care or a documented absence of a required system element. Holes were identified through triangulation of at least two independent data sources (e.g., surveillance records confirmed by field observations; focus group findings confirmed by epidemiological reports). The Group Model Building workshop served as a participatory validation exercise, although no actual scoring took place. The 69 stakeholders (including frontline health workers, veterinarians, municipal officials, and academic researchers) collectively mapped barriers and, in this process, confirmed and added new gaps to the system vulnerabilities identified in our data^19^. While the Swiss cheese model as applied here is conceptual rather than quantitative, we provide a structured summary table (Supplementary Table S1) that lists each hole, the data sources supporting it, and the layer to which it belongs, enabling readers to assess the evidence base for each identified vulnerability.

### Study area

**Fig. 8:**
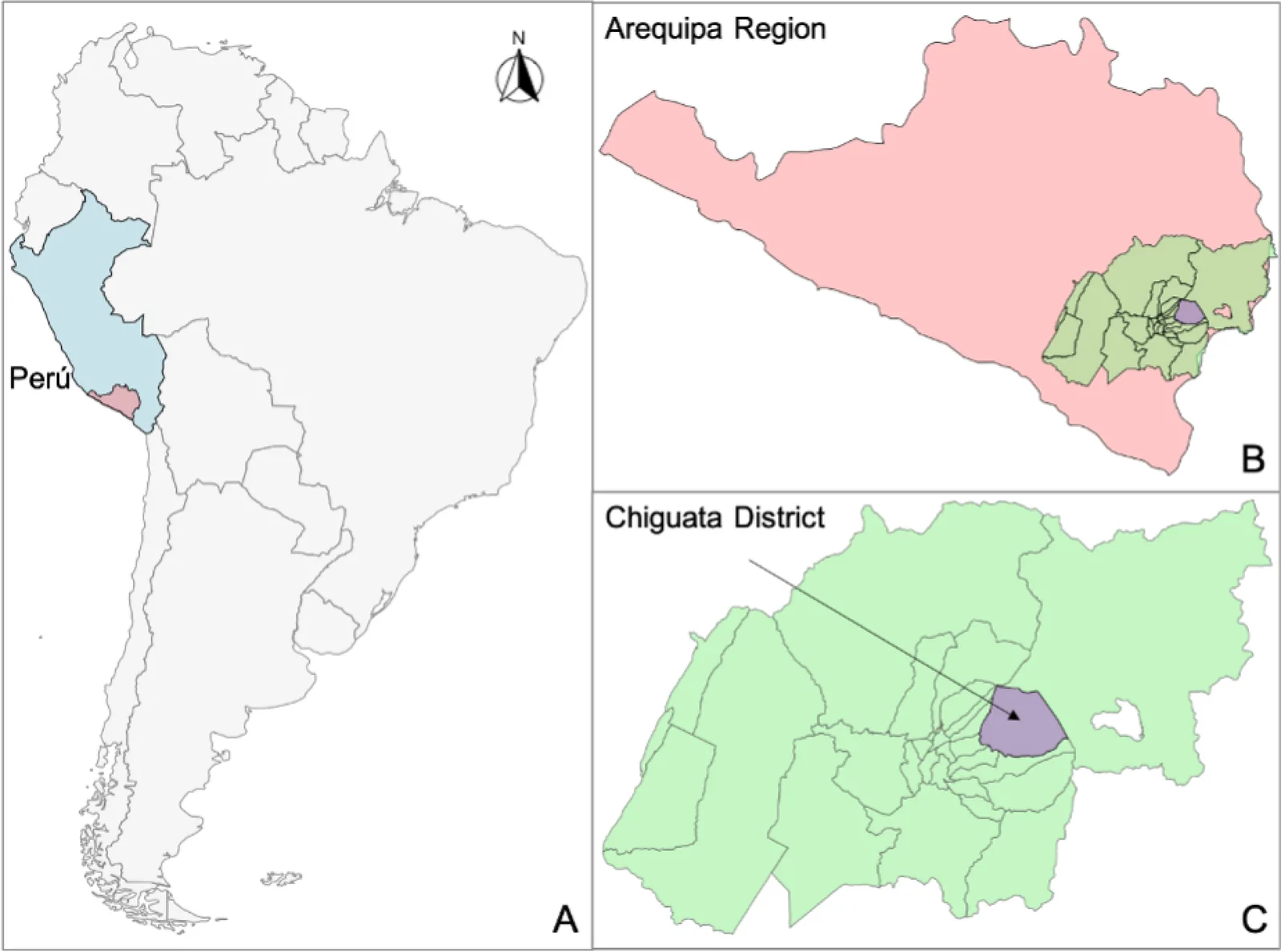
(**A**) Map of Peru showing the national location of the 2023 human rabies case. (**B**) Arequipa region, an area endemic for canine rabies. (**C**) Location of Chiguata district (purple) within the metropolitan area of Arequipa, alongside districts reporting at least one canine rabies case (green). All maps were generated in R version 4.2.1^45^ using publicly available shapefiles from the *rnaturalearth* package and the Instituto Nacional de Estadística e Informática (INEI), Peru.

The study was conducted in the city of Arequipa, Peru. The focal area for the 2023 human rabies case was the district of San Bernardo de Chiguata (Chiguata hereon), a rapidly expanding peri-urban area (Fig. 8), characterized by mixed agricultural and residential land use, high human mobility, and the presence of free-roaming and feral dogs populations. Chiguata is connected to other districts reporting active canine rabies transmission and contains ecological features such as dry water channels that facilitate dog movement across urban and peri-urban areas^44^.

### Data source and collection

We compiled data from four complementary sources collected between 2015 and 2023:

#### Epidemiological surveillance data

We obtained canine rabies surveillance data from the MOH records (Fig. 2S) for the period 2015–2013. Variables extracted included number of samples submitted for rabies testing, confirmed canine rabies cases, geographic location of cases, and reporting timeliness. Canine rabies cases were defined per Peru’s national surveillance protocol^26^: a confirmed case required laboratory detection of rabies virus antigen via direct fluorescent antibody test (dFAT) on brain tissue submitted by regional health authorities. Samples were obtained from animals presenting clinical signs of rabies or involved in biting incidents.

We also reviewed epidemiological investigation reports generated following the confirmation of the 2023 human rabies case^20,21,44^, including exposure history, health-care seeking trajectory, and timing of diagnosis.

To evaluate temporal trends in surveillance intensity, we calculated annual counts of samples submitted for laboratory confirmation and compared these trends with reported canine rabies cases. Data completeness is inherently limited by passive surveillance dynamics: sample submission depends on field reporting by community health workers and veterinary officers, both of which are subject to resource constraints and geographic accessibility. We quantified this limitation by calculating the annual ratio of samples submitted relative to confirmed cases, which declined by approximately 80% between 2015 and 2020 (Fig. 4), indicating substantial underdetection during this period. Human population denominators were obtained from national census projections^46^; canine population denominators were estimated from our annual door-to-door surveys using household-level dog ownership data, as described in the “Dog population and vaccination data” section. We acknowledge that both denominators carry uncertainty, particularly in rapidly expanding peri-urban areas such as Chiguata, where household mobility, and the potential underestimation of free-roaming dog populations introduce measurement error.

#### Dog population and vaccination data

Dog population size and vaccination coverage were estimated using repeated door-to-door household surveys conducted annually between 2016 and 2023 in districts affected by canine rabies transmission. Surveys collected information on dog ownership, number of dogs per household, vaccination status, participation in MDVCs, and household demographic characteristics. Human-to-dog ratios were calculated using household-level data and compared with official ratios used for vaccination planning. Vaccination coverage was estimated as the proportion of dogs reported vaccinated during MDVCs relative to estimated dog population size.

#### Dog ecology and movement patterns

To characterize the ecological dynamics of free-roaming dogs, we incorporated data from GPS tracking studies conducted in Arequipa between 2016 and 2020 to characterize free-roaming dog movement patterns^15^ complemented by field observations documenting feral dog populations inhabiting caves and water channels in peri-urban areas^44^. Observational data included photographic documentation and geographic location of feral dog aggregations.

#### Qualitative and stakeholder data

We analyzed qualitative data derived from three study populations. Fifty-six adult female dog owners from urban (n = 31) and peri-urban (n = 25) areas participated in seven focus group discussions, recruited through purposive door-to-door sampling; thematic saturation was reached after five focus group discussions, and two additional groups confirmed no new themes emerged. Women were selected for participation based on our previous work in Arequipa, which found that they were typically the primary caregivers for household pets and were most knowledgeable about household management practices. Door-to-door (D2D) surveys of up to 8,000 households annually, between 2016 and 2023, to assess perceptions of rabies risk, barriers to accessing PEP, and participation in MDVCs. Discussions were audio recorded, transcribed, and coded using thematic analysis to identify recurrent barriers to prevention behaviors. Descriptions of the study communities and the data collection approaches are detailed elsewhere^3,23,25^. Sixty-nine MDVC implementers and authorities representing the 20 health networks participated in a day-long group model building workshop focused on reflecting on past MDVCs and co-designing improvements to the system. At one point during the workshop, multidisciplinary teams were divided into 14 working groups of 4–5 individuals (implementers) and one with the 12 attending authorities. Structured field observations were conducted by a dedicated research manager across 41 MDVCs between June and October 2022 and documented in a digital field notebook. All focus group and working group recordings were transcribed verbatim in Spanish. All focus and working group transcripts, as well as the field observations, were then double-coded and analyzed in Dedoose (v9.0.90) using a codebook developed based on the Consolidated Framework for Implementation Research (CFIR) domains and subdomains as main themes and sub-categories, respectively. Two team members independently double-coded all transcripts; discrepancies were resolved through structured discussion. Additionally, community focus group findings were stratified by setting (urban vs. peri-urban). The methodological details of this workshop are described elsewhere^19^.

#### Stakeholder mapping analysis

Stakeholder mapping was conducted to characterize the governance structure of rabies control in Peru and identify gaps in multisectoral coordination. Institutional actors were categorized according to level of influence, operational role, and directionality of communication flows. Relationships between actors were classified as hierarchical, collaborative, or absent. Stakeholders were grouped into four clusters:

1. national regulatory authorities
2. regional and local health authorities
3. municipal actors
4. peripheral stakeholders

Mapping outputs were visually represented to identify structural connections or disconnections between sectors responsible for the various components of Peru’s human rabies elimination strategy.

### Ethics

The study was conducted under IRB approval from University of Pennsylvania (UPENN), Universidad Peruana Cayetano Heredia (UPCH) and Tulane University for conducting focus groups and surveys (IRB # 823736 and 850695, 65369 and 207735, 606720 and 2022-1134, respectively), and workshops and observations of dog rabies vaccination campaigns in Arequipa (IRB 823736, 65369, 606720, respectively). All participants in focus groups and household surveys provided informed consent prior to participation. All procedures were conducted in accordance with relevant ethical guidelines and regulations.

## Supplementary Information

Below is the link to the electronic supplementary material.

Supplementary Material 1

Supplementary Material 2

## Data Availability

The datasets generated and analyzed during the current study are available from the corresponding author upon request.
