# Supplementary material for: A one health analysis of the reemergence of dog-mediated human rabies in Peru
Source: Sci Rep. 2026 Jul 31;16:36695. doi: 10.1038/s41598-026-65032-3 (PMC13727665; doi:10.1038/s41598-026-65032-3)
Supplement: Supplementary file 1 [file 41598_2026_65032_MOESM1_ESM.docx]

Supplementary

**Figure 1S.** *Torrentera* during the dry season, with waste disposal in it. Photograph by UPCH research team, 2023.

**Figure 2S.** Dog vaccination coverage in Arequipa, Perú 2015 - 2023.
