# Supplementary material for: A one health analysis of the reemergence of dog-mediated human rabies in Peru
Source: Sci Rep. 2026 Jul 31;16:36695. doi: 10.1038/s41598-026-65032-3 (PMC13727665; doi:10.1038/s41598-026-65032-3)
Supplement: Supplementary file 2 [file 41598_2026_65032_MOESM2_ESM.docx]

**Supplementary Table S1.** Swiss Cheese Model—Identified system holes, supporting evidence, and severity assessment.

| **#** | **Layer** | **System hole (documented vulnerability)** | **Expected state / standard of care** | **Supporting data source(s)** | **Severity*** |
| --- | --- | --- | --- | --- | --- |
| **H1** | **1. Policies** | 80% decline in canine surveillance sampling (2015–2020), leading to massive underdetection of circulating rabies virus | Annual surveillance sampling maintained at levels sufficient to detect ongoing transmission per MINSA Technical Norm (26) | MOH official surveillance records (Figure 4); epidemiological reports (18–20) | **High** |
| **H2** |  | Outdated Human-to-Dog (H:D) ratio used for vaccination planning (official ratio 10:1; survey-derived ratio 6:1), resulting in chronic underestimation of the dog population | Dog population denominator updated annually using field-based enumeration methods (26) | Door-to-door household surveys 2016–2023 (21); official planning records (Table 1) | **High** |
| **H3** |  | High inter-annual variability in H:D ratio estimates (range 2.5–3.4 across consecutive years), preventing reliable longitudinal tracking of vaccination coverage | Consistent, reproducible denominator estimation methodology applied across years | Official network planning records (Table 1); door-to-door surveys (21) | **Moderate** |
| **H4** |  | Reported MDVC coverage exceeding 100% in Chiguata (113.2% in 2022), indicating unreliable coverage metrics used for decision-making | Coverage metrics validated against independent population estimates; >100% triggers denominator review | Chiguata network official records (Table 1); door-to-door surveys | **Moderate** |
| **H5** |  | No formal assigned institutional responsibility for feral dog population management; MOH manages vaccines while municipalities manage environment, with no sector accountable for unowned dogs | Clear legal mandate and budget line for feral dog management assigned to a specific institutional actor (24, 34) | Stakeholder mapping (Figure 7); GMB workshop data (19); national regulatory review (26, 27, 34) | **High** |
| **H6** |  | Absence of formal horizontal coordination mechanism between MOH/GERESA and municipal governments, creating governance fragmentation in environmental risk management | Formal inter-sectoral coordination protocol between health and municipal actors for rabies control | Stakeholder mapping (Figure 7); GMB workshop (19) | **High** |
| **H7** | **2. Outbreak response** | Approximately 62% of canine rabies cases in the region suffered from late outbreak reports, delaying reactive control measures (ring vaccination, PEP administration) | All confirmed canine rabies cases reported and investigated within 24–48 hours per MINSA protocol | Timeliness analysis of outbreak reporting (22) | **High** |
| **H8** |  | Clandestine burial of rabid dogs by community members to avoid social stigma, masking the true extent of the outbreak and preventing laboratory confirmation | All suspected rabid animals reported and submitted for diagnostic testing; no community barriers to reporting | Community focus groups (n=56 participants, 7 groups); door-to-door surveys (23) | **High** |
| **H9** |  | Distrust of official vaccination teams by community members, contributing to sub-optimal MDVC participation and reduced herd immunity in affected areas | Community trust in health promotion teams sufficient to achieve ≥70% MDVC coverage in all districts | Community focus groups; door-to-door surveys 2016–2023 (3, 23); GMB workshop (19) | **Moderate** |
| **H10** |  | Peripheral exclusion of high-influence actors (SERFOR, Police/security forces) from active surveillance and outbreak response, representing missed enforcement and reporting capacity | All relevant institutional actors with surveillance or enforcement capacity formally integrated into outbreak response protocols | Stakeholder mapping (Figure 7); GMB workshop (19) | **Moderate** |
| **H11** | **3. System capacity building** | Clinical misdiagnosis of the 2023 victim: rabies symptoms misattributed to generalized anxiety and psychiatric disorders across multiple health center visits, delaying correct diagnosis until the disease was irreversible | Rabies included in differential diagnosis for all patients presenting with encephalitic or progressive neurological symptoms with history of potential animal exposure in endemic areas (26) | Epidemiological investigation reports (refs 18–20); case timeline (Figure 1) | **High** |
| **H12** |  | Absence of routine inquiry about animal exposure history by clinical providers in peri-urban settings, preventing early identification of rabies exposure | Standard clinical intake protocol includes animal exposure history for all patients in canine-rabies-endemic districts | Epidemiological investigation reports (18–20); GMB workshop data (19) | **High** |
| **H13** |  | No rabies vaccine available at the Chiguata health post at the time of the incident; victim referred to a facility 11 km away through mountainous terrain, creating delays in PEP access | Post-exposure prophylaxis (PEP) available at or within 5 km of all communities with documented canine rabies transmission (26) | Epidemiological investigation reports (18–20); Figure 5 (health center distribution map) | **High** |
| **H14** |  | Low community risk perception: victim did not report the dog bite, consistent with normalization of dog bites in peri-urban Arequipa and documented barriers to care-seeking | Community members in endemic areas recognize dog bites as a medical emergency and seek care promptly | Community focus groups; door-to-door surveys (3, 23); epidemiological reports (18–20) | **High** |
| **H15** |  | Absence of a structured community education plan in Chiguata at the time of the 2023 case; no sustained inter-outbreak communication strategy to maintain risk awareness | Continuous (not campaign-only) community health education on rabies risk maintained in all districts with active canine transmission | Community focus groups (n=56); GMB workshop (19); field observations (41 MDVCs, 2022) | **Moderate** |
| **H16** |  | Decline in clinical training continuity during inter-outbreak periods; no mechanism to sustain provider competency in rabies recognition between human case events | Annual clinical refresher training on rabies recognition and PEP protocols mandatory for all providers in endemic districts | GMB workshop data (28); epidemiological case reports (18–20) | **Moderate** |
| **H17** | **4. Socio-geographical factors** | Torrenteras (dry water channels) serve as unmonitored ecological corridors connecting feral dog populations in peri-urban areas (including Chiguata) with urban districts, enabling undetected viral movement outside vaccination campaign boundaries | Ecological corridors facilitating dog movement identified, monitored, and incorporated into MDVC geographic planning | GPS tracking studies 2016–2020 (15); field observations and photographic documentation (Figure 6); torrentera spatial analysis (12) | **High** |
| **H18** |  | Feral dog populations inhabiting caves and torrenteras remain entirely outside the reach of traditional vaccination campaigns, constituting an unvaccinated reservoir of unknown size | Dog population surveys include enumeration of unowned/feral dogs; vaccination strategies adapted to reach unowned populations | Field observations (Figure 6A); GPS tracking studies (15); MINSA surveillance data (24) | **High** |
| **H19** |  | High household mobility and frequent recent in-migration in Chiguata associated with lower vaccination coverage and delayed care-seeking, creating pockets of susceptibility not captured by aggregate coverage metrics | Vaccination coverage analyses account for population mobility and in-migration; mobile populations specifically targeted in MDVC planning | Field observations (41 MDVCs, 2022); door-to-door surveys (13, 23); epidemiological reports | **Moderate** |
| **H20** |  | Seasonal and economic migration between Arequipa and Puno, consistent with the reintroduction of the rabies virus prior to its detection in 2015, representing an ongoing cross-jurisdictional transmission risk not addressed in current elimination planning | Cross-jurisdictional rabies surveillance and vaccination coordination between Arequipa and neighboring endemic regions | Genomic epidemiology study (25); field observations | **Moderate** |

*** Severity rating (heuristic, consensus-based): High** = documented, persistent vulnerability with direct pathway to human exposure; **Moderate** = documented vulnerability that amplifies risk but requires co-occurrence with other holes; **Low** = documented vulnerability with indirect or uncertain contribution. Ratings were assigned through research-team consensus and validated against GMB workshop findings.

**Abbreviations:** MDVC, mass dog vaccination campaign; MOH, Ministry of Health; GERESA, Regional Health Government; GMB, Group Model Building; PEP, post-exposure prophylaxis; MINSA, Ministry of Health of Peru; SERFOR, National Forestry and Wildlife Service; H:D, Human-to-Dog ratio.
